# Synthesis and Biological Activities of Camphor Hydrazone and Imine Derivatives

**DOI:** 10.3390/scipharm84030467

**Published:** 2015-10-18

**Authors:** Emerson T. da Silva, Adriele da Silva Araújo, Adriana M. Moraes, Leidiane A. de Souza, Maria Cristina Silva Lourenço, Marcus V. N. de Souza, James L. Wardell, Solange M. S. V. Wardell

**Affiliations:** 1 Fundação Oswaldo Cruz (Fiocruz), Instituto de Tecnologia em Fármacos Farmanguinhos, Rua Sizenando Nabuco, 100, Manguinhos 21041-250, Rio de Janeiro, Brazil; esilva@far.fiocruz.br (E.T.d.S.); adriele.cp2@gmail.com (A.d.S.A.); m_drik@yahoo.com.br (A.M.M.); araujo.leidiane91@gmail.com (L.A.d.S.); j.wardell@abdn.ac.uk (J.L.W.); 2 Instituto de Pesquisa Clínica Evandro Chagas (IPEC)Av. Brasil, 4365, Fiocruz, 21040-900 Rio de Janeiro, Brazil; cristina.lourenco@ipec.fiocruz.br; 3 Department of Chemistry, University of Aberdeen, Aberdeen AB2 3UE, Scotland, UK; 4 CHEMSOL, 1 Harcourt Road, Aberdeen AB15 5NY, Scotland, UK; solangewardell@gmail.com

**Keywords:** camphor, sonochemistry, hydrazones, imines, tuberculosis

## Abstract

Both sonochemical and classical methodologies have been employed to convert camphor, 1,7,7-trimethylbicyclo[2.2.1]heptan-2-one, C_9_H_16_C=O, into a number of derivatives including hydrazones, C_9_H_16_C=N-NHAr **3**, imines, C_9_H_16_C=N-R **7**, and the key intermediate nitroimine, C_9_H_16_C=N-NO_2_ **6**. Reactions of nitroamine **6** with nucleophiles by classical methods provided the desired compounds in a range of yields. In evaluations of activity against *Mycobacterium tuberculosis*, compound **7j** exhibited the best activity (minimal inhibitory concentration (MIC) = 3.12 µg/mL), comparable to that of the antitubercular drug ethambutol. The other derivatives displayed modest antimycobacterial activities at 25–50 µg/mL. In in vitro tests against cancer cell lines, none of the synthesized camphor compounds exhibited cytotoxic activities.

## 1. Introduction

Camphor, 1,7,7-trimethylbicyclo[2.2.1]heptan-2-one, is a natural product used since antiquity in a wide range of applications, such as in food flavourings, fumigants, perfumes, cosmetics, household cleaners, and topically applied analgesics [1]. Natural camphor occurs in dextrorotatory form, i.e., *R*,*R*-(+)-camphor, and is obtained through distillation of the wood from the camphor laurel tree (*Cinnamomum camphora*) or by chemical transformation of other natural products, such as turpentine [1]. The laevorotatory form (*S*,*S*-Camphor) exists only in a synthetic form or in very little quantities in specific species of plants [2]. In chemistry, camphor has been used as a selector in chiral separations [3], as a shift reagent in nuclear magnetic resonance (NMR) [4], as a chiral auxiliary [5], and as part of a catalyst system [6] as well as a versatile chemical precursor [7]. In the medical chemistry field, studies have revealed that camphor possesses a range of useful biological activities, being an antiviral, antimicrobial, antitussive, and analgesic agent [8].

Despite the general importance of camphor and its derivatives in several fields, few antitubercular and anticancer studies have been described. There are very few reports on the use of sonochemistry in synthesizing camphor derivatives. To address the lack of information in these areas, we have conducted a comparative study of the uses of sonochemistry and classical methodologies in synthesizing camphor derivatives and an evaluation of these derivatives as potential antitubercular and anticancer agents.

## 2. Results and Discussion

### 2.1. Chemistry

**Scheme 1:**
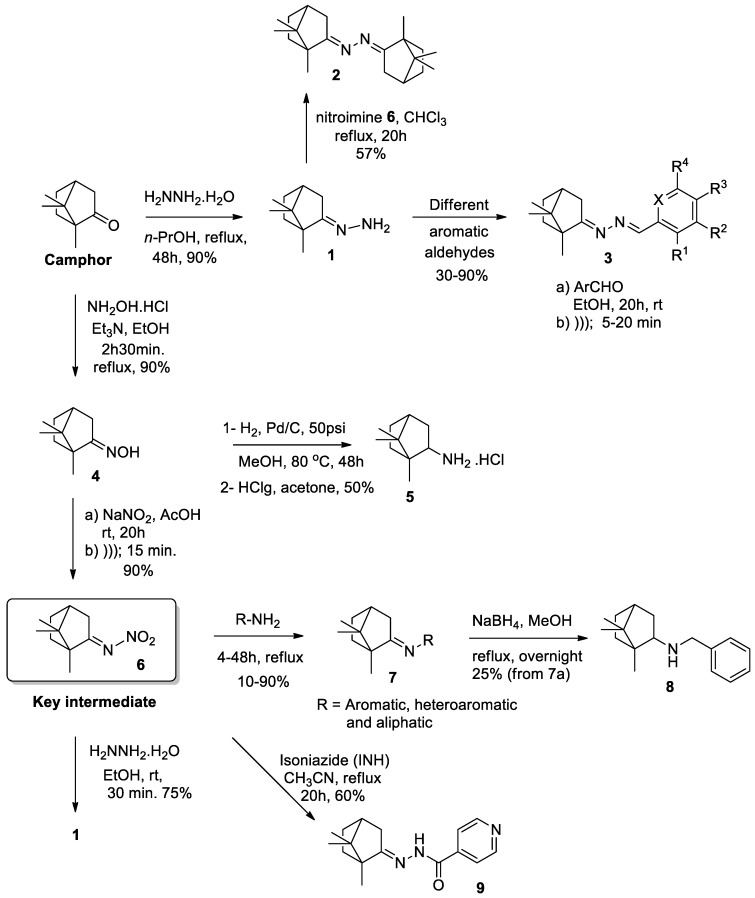
General synthetic scheme for the camphor derivatives.

The camphor derivatives were synthesized as shown in Scheme 1. Hydrazine **1**, prepared from camphor and hydrazine hydrate (80%) in 90% yield, was coupled with different arenecarbaldehydes to produce **3** in 30%–90% yields. For the preparation of the series **3**, sonochemistry was found to be far superior to classical methodologies; in particular by drastically reducing the reaction time from ca. 20 h to a few minutes: only in the sonochemical synthesis of compound **3e** was a reaction time greater than one hour required, see Table 1. The oxime **4**, synthesized from hydroxylamine hydrochloride in 90% yield, was reduced to the amine hydrochloride **5** in a 50% yield. Comparison of the reaction conditions for the synthesis of compound **6** from oxime **4** and sodium nitrite again showed the advantages of using a sonochemical approach. Compound **6** was a key intermediate due to its ready reaction by classical means with various nucleophiles, such as hydrazine and amines, to give compounds, **1**, **7**, and **9** (see Scheme 1). Yields of compounds **7** varied from 10% to 90%. It is of interest to note that ultrasonic irradiation was clearly inferior to classical methodologies in syntheses involving the nitroamine **6**, although, as indicated above, compound **6** could be produced from **4** by both sonochemical and classical methodologies. Reduction of **7a** to **8** occurred upon reaction with sodium borohydride.

All compounds were characterized by ^1^H-NMR, ^13^C-NMR, and infrared (IR) spectra as well as by mass spectrometry (MS) data, and additionally in the case of compound **3k** by X-ray crystallography. The chemical shifts, multiplicities, and coupling constants in the ^1^H-NMR spectra confirmed the proposed structures. In general, for compounds **3**, the ^1^H-NMR spectra showed the characteristic signal for the N=CH proton at 8.19–8.80 ppm. Furthermore, the IR spectra showed N=C stretching vibrations at 1624–1661 cm^−1^. The chemical shifts of all aliphatic and aryl protons occurred in the regions normally found. Compound **9**, as is typical of acylhydrazones and as shown by the ^1^H-NMR spectrum, exists as an E_N=C_/E_C(O)NH_ and E_N=C_/Z_C(O)NH_ mixture (in a 5:1 ratio) of conformers in solution [9]. The IR spectrum of **9** showed a C=O stretching vibration at 1638 cm^−1^.

### 2.2. Crystallography

A number of derivatives, **3**, were considered as candidates for structure determinations by X-ray crystallography. However, poor crystals or powders were generally obtained upon recrystallization from common organic solvents: the best crystals were obtained for **3k** from solution in 2-MeOCH_2_CH_2_OH. Moreover, even here, the crystals were not great and the best refinement was only about 12%. However, the atom connections, bond lengths, and angles within the hydrazonyl side chain were securely determined.

**Figure 1:**
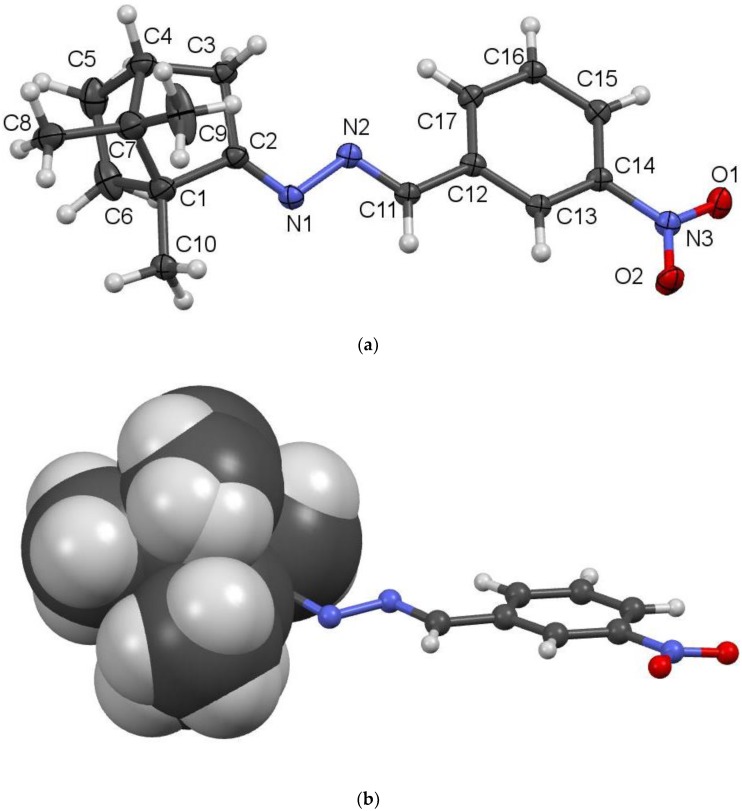
(**a**) The atom arrangements and numbering scheme for molecule **3k**: only the major form of the highly disordered camphor group has been illustrated. Probability ellipsoids have been drawn at the 50% level; Hydrogen atoms have been drawn as spheres of arbitrary radius, (**b**) a version of the molecule in which the camphor group atoms were drawn in space filling forms and the N-N=CHC_6_H_4_NO_2_-*m* was drawn in ball-and-stick forms.

The crystal structure was solved from data collected at 120(2)K [10,11,12]. The asymmetric unit consisted of a single molecule. The aliphatic carbon atoms within the camphor moiety were highly disordered: Figure 1a shows the atom arrangements and numbering scheme for the predominant form. The atoms in the side chain of the camphor fragment, C=N-NH-C_6_H_4_-NO_2_-*m*, experienced no disorder, with precise bond angles and lengths. This side chain is not completely planar, as indicated by the angles between the N1-N2-C11 chain and the nitro group with the attached benzene ring of 5.1(1) and 12.1(2)°, respectively. Due to the disorder of the camphor moiety, the C=N-NH-C_6_H_4_-NO_2_-*m* side chain appears to emerge from a hydrocarbon ball: an attempt to give an impression of this is shown in Figure 1b, in which space-filling forms have been used for the atoms in the camphor fragment. The molecule clearly has two extreme poles—a polar end and a non-polar end. Among our recent studies have been investigations of the structures of various biologically active hydrazones of the type heteroaryl-NH-N=CH-Ar, such as heteroaryl = -7-chloroquinolin-4-yl [13], quinoxalin-2-yl [14], 1,3-benzothiazol-2-yl [15], and 5-phenyl-1,3,4-thiadiazol-2-yl [16]. In all these cases, the heteroaryl groups are planar or nearly so and are thus much different sterically from the bulky, non-planar camphor unit. Such differences should affect the biological activities, solubilities, etc.

Not drawn in Figure 1a is the intramolecular C6-H6A---N1 hydrogen bond. Bond angles and lengths in the C1-N1-N2-C11-C12 fragment and other selected parameters are listed in Table 2. As found in other hydrazones, the bond lengths in the hydrazonyl fragment indicate considerable electron delocalization [13,14,15,16]. The geometry of the (camphor)-C1-N1-N2-C11-C12 fragment is (*E*,*E*). PLATON analysis indicated that molecules of **3k** are linked into a 3-dimensional array via combinations of π(phenyl)-π(phenyl) stacking interactions and C3-H3A---O1*^i^*, C3-H3B---O1*^ii^* and C15-H15---N1*^iii^* intermolecular hydrogen bonds (symmetry operations: *i* = x, 1/2 + y, ½-z; *ii* = −x, −1 − y, 1 − z; *iii* = −x, −1/2 + y, 1/2 − z) [17]. In this arrangement, the polar N-NH-C_6_H_4_-NO_2_-*m* chains are engulfed by the non-polar camphor balls, see Figure 1b.

### 2.3. Antimycobacterial Activity against *Mycobacterium tuberculosis* H37Rv (ATCC 27294)

The antimycobacterial activities of the synthesized compounds were assessed against *M. tuberculosis* ATCC 27294 [18] using the microplate Alamar Blue assay (MABA) [19] (Table 2 and Table 3). This methodology is nontoxic, uses a thermally-stable reagent and shows good correlation with proportional and BACTEC radiometric methods [20,21].

The antimycobacterial activities of hydrazones **3** and imines **7** are shown in Table 2 and Table 3. It is noticeable that the majority of moderately active compounds **3**, i.e., those having minimal inhibitory concentration (MIC) values equal to or less than 50 µg/mL, have *ortho*-sited substituents in the aryl ring: the most active is **3p** (MIC value = 25 µg/mL) with an *ortho* nitro group. Among the other moderately active compounds are **3n** (*o*-Br), **3o** (*o*-OH), and **3v** (*o*-OH-*p*-OMe). These substituents, OH, OMe, Br, and O_2_N, exhibit a range of electronic effects and spatial needs. There is no apparent link between the activities and properties these substituents possess, so different mechanisms of activity are suggested. From our earlier studies and others reported in the literature on the biological activities of hydrazones and heteroaryl-NH-N=CH-Ar (e.g., heteroaryl = 2-quinoxalinyl, etc.) [14], compounds having an *ortho*-hydroxyl aryl group, such as Ar = *o*-HOC_6_H_4_, were found to be particularly active. This activity was assigned to their ability to act as *N*,*N*,*O*-tridentate ligands in chelating essential metals such as iron, copper, and zinc. A similar role has been suggested for heteroaryl-NH-N=CH-pyridin-2-yl compounds. The derivatives, **3o** and **3v**, as well as **3x**, however, at best can only operate as bidentate chelators which greatly reduce their ability to sequester any essential metal.

In a more limited study of the imines **7**, the most active compound by far, with an MIC value of 3.12 µg/mL, was **7j** with an *o*-hydroxyl group. This is comparable activity to that of the antitubercular drug ethambutol. No other compound **7** had an activity anywhere near that of **7j**.

The isoniazid derivative **9** exhibited a moderate activity (50 µg/mL) while compounds **1**, **2**, and **4**–**6** did not exhibit antimycobacterial activity.

### 2.4. Cytotoxicity against Cancer Cell Lines

All compounds were tested in vitro against three human cancer cell lines: SF-295 (glioblastoma), OVCAR-8 (ovary), and HCT-116 (colon) (National Cancer Institute, Bethesda, MD, USA) at 5 μg/mL using the MTT assay. Unfortunately, none of the compounds prepared in this study displayed cytotoxicity activities.

### 2.5. Toxicity Risks

Only compounds **3r**, **3v**, **3x**, **6**, **7i**, and **9** were indicated by the OSIRIS Property Explorer program [22] to possess potential toxicity risks.

## 3. Materials and Methods

Melting points were determined with a Fisatom 130 apparatus (Fisatom, São Paulo, Brazil) and are uncorrected. NMR spectra were determined using 400 or 500 MHz Bruker AC spectrometers (Karlsruhe, Germany). Infrared spectra were obtained using a Perkin-Elmer 467 FTIR spectrometer (Cleveland, OH, USA) with samples in potassium bromide disks. Mass spectra were recorded on the Agilent 122 5532 GC/MS column by electron impact (Santa Clara, CA, USA). The progress of the reactions was monitored by thin-layer chromatography (TLC) on 2.0 cm × 6.0 cm aluminum sheets (silica gel 60, HF-254, Merck, Kenilworth, NJ, USA) with a thickness of 0.25 mm, using ultraviolet light irradiation. For column chromatography, Merck silica gel (230–400 mesh) was used. Solvents and reagents were used without further purification. An ultrasonic homogenizer model Desruptor Eco Sonics (25 KHz, Eco Sonics, Indaiatuba, Brazil) was used to carry out the reactions at 90 W. Arenealdehydes and *D*-camphor (*R*,*R-*(+)-camphor) were obtained from commercial sources. Oxime **4** and nitroimine **6** were prepared from *D*-camphor following published procedures [23].

### 3.1. Synthesis of Nitroimine (**6**) Using Ultrasonic Irradiation

To a solution of the oxime **4** (4.32 g, 26.23 mmol) in acetic acid (130 mL), an aqueous solution of sodium nitrite was added (65 mL, 10%). The reaction mixture was irradiated with ultrasonic energy until TLC indicated the complete consumption of **4** (15 min). After cooling at 0 °C, a solution of NaOH (150 mL, 50%) was added and the mixture was extracted with ethyl acetate (4 × 50 mL). The combined organic phases were dried over anhydrous Na_2_SO_4_ and concentrated under reduced pressure to furnish **6** as an oil (90% yield, 4.11 g).

### 3.2. Synthesis of (E)-(1,7,7-Trimethylbicyclo[2.2.1]heptan-2-ylidene)hydrazine (**1**). 

To a solution of camphor (3.0 g; 19.74 mmol) in *n*-propanol (50 mL) was added hydrazine hydrate (10 equivalents, 12.5 mL, 80%). The mixture was refluxed until TLC indicated completed consumption of the camphor (48 h). The reaction mixture was concentrated under vacuum and successively added (30 mL) to the residue water with stirring, along with ethyl acetate (30 mL). The organic layer was collected. The aqueous phase was extracted with ethyl acetate (2 × 30 mL) and the combined organic phases were dried over anhydrous Na_2_SO_4_ and concentrated under reduced pressure to leave a solid, melting point (mp): 50–53 °C (lit. [24] mp: 54–55 °C).

### 3.3 Synthesis of (1E,2E)-1,2-Bis(1,7,7-trimethylbicyclo[2.2.1]heptan-2-ylidene)hydrazine (**2**). 

To a solution of the hydrazine **1** (0.202 g; 1.22 mmol) in CHCl_3_ (10 mL) nitroimine **6** was added (0.239 g; 1.22 mmol). The mixture was refluxed until TLC indicated the consumption of compound **6**. An aqueous solution of Na_2_CO_3_ (10%, 20 mL) was added with stirring and the phases were separated. The aqueous phase was extracted with CHCl_3_ (2 × 10 mL). The combined organic phases were dried over anhydrous Na_2_SO_4_ and concentrated under reduced pressure. The crude residue was purified by column chromatography on silica gel (SiO_2_, 70–230 mesh, AcOEt/*n*-Hex 10%) to afford the desired product in 57%, mp: 180–183 °C (lit. [25] mp: 186–187 °C). ^1^H-NMR (CDCl_3_, 400 MHz) δ: 2.15–2.35 (2H, m, N=C-CH_2a_), 1.15–1.95 (12H, m, 2CH, 2-N=C-CH_2b_, 2-CH_2_-CH_2_), 1.03 (6H, s, 2-CH_3_), 0.92 (6H, s, 2-CH_3_), 0.79 (6H, s, 2-CH_3_). ^13^C-NMR (CDCl_3_, 100 MHz) δ: 175.96, 175.90, 52.45, 52.44, 43.82, 48.24, 47.64, 46.96, 43.82, 34.96, 18.58, 18.52, 17.71, 17.67, 10.14, 10.12. IR (KBr) v, cm^−1^: 1669 (C=N). GC: 100% MS: (*m*/*z*, %): 300 (M^+^, 90), 231 (63), 150 (61), 109 (41).

### 3.4. General Procedures for the Synthesis of Camphor Hydrazone Derivatives (**3**)

(a) Without Sonication

To a solution of hydrazine **1** (0.25 g; 1.5 mmol) in ethanol (10 mL) was added the appropriate arenealdehyde (1 or 0.8 eq.). The reaction mixture was stirred for 20 h when TLC indicated the consumption of the aldehyde. The hydrazone product was normally obtained pure after filtering and washing with cold ethanol or diethyl ether (5 to 10 mL). However, in some cases trituration or column chromatography was necessary, as indicated below.

(b) With Sonication

Similar amounts of reagents and solvents were used as in (a). The reaction mixture was irradiated with an ultrasonic probe until TLC indicated the reaction was complete. With the exception of the reaction producing **7e**, all other reactions were complete in 5–18 min.

*(1E,2E)-1-Benzylidene-2-(1,7,7-trimethylbicyclo[2.2.1]heptan-2-ylidene)hydrazine* (**3a**). Purified by column chromatography on silica gel (70–230 mesh, AcOEt/*n*-Hex 5%). Yield: 64%, oil. ^1^H-NMR (CDCl_3_, 400 MHz) δ: 8.33 (1H, s, CH=N), 7.30–7.80 (5H, m, phenyl), 2.10–2.75 (2H, m, N=C-CH_2_), 1.94 (1H, t, CH, *J* = 4.28 Hz), 1.25–1.92 (4H, m, CH_2_-CH_2_), 1.10 (3H, s, CH_3_), 0.96 (3H, s, CH_3_), 0.84 (3H, s, CH_3_). ^13^C-NMR (CDCl_3_, 100 MHz) δ: 182.77, 157.70, 134.96, 130.68, 128.81, 128.29, 52.88, 48.13, 44.12, 36.21, 32.87, 27.52, 19.85, 19.02, 11.50. IR (KBr) v, cm^−1^: 1650 (C=N). HRMS (ESI, +H): Theoretical mass calculated for [C_17_H_22_N_2_ + H]: 255.1861; Found: 255.1856.

*(1E,2E)-1-(4-Fluorobenzylidene)-2-(1,7,7-trimethylbicyclo[2.2.1]heptan-2-ylidene)hydrazine* (**3b**). Purified by column chromatography on silica gel (70–230 mesh, AcOEt/*n*-Hex 10%). Yield: 80%, mp: 123–125 °C. ^1^H-NMR (CDCl_3_, 400 MHz) δ: 8.31 (1H, s, CH=N), 7.00–7.76 (4H, m, phenyl), 2.15–2.67 (2H, m, N=C-CH_2_), 1.94 (1H, t, CH, *J* = 4.28 Hz), 1.25–1.95 (4H, m, CH_2_-CH_2_), 1.10 (3H, s, CH_3_), 0.97 (3H, s, CH_3_), 0.84 (3H, s, CH_3_). ^13^C-NMR (CDCl_3_, 100 MHz) δ: 180.76, 164.66, 162.19, 155.86, 130.98, 129.99, 115.84, 52.21, 47.33, 43.18, 35.34, 32.25, 26.73, 19.25, 18.84, 11.23. IR (KBr) v, cm^−1^: 1624 (C=N). HRMS (ESI, +H): Theoretical mass calculated for [C_17_H_21_FN_2_ +H]: 273.1767; Found: 273.1765.

*(1E,2E)-1-(4-Chlorobenzylidene)-2-(1,7,7-trimethylbicyclo[2.2.1]heptan-2-ylidene)hydrazine* (**3c**). Purified by column chromatography on silica gel (70–230 mesh, AcOEt/*n*-Hex 10%). Yield: 71%, oil. ^1^H-NMR (CDCl_3_, 400 MHz) δ: 8.30 (1H, s, CH=N), 7.35–7.75 (4H, m, phenyl), 2.15-2.71 (2H, m, N=C-CH_2_), 1.94 (1H, t, CH, *J* =4.24 Hz), 1.20–1.95 (4H, m, CH_2_-CH_2_), 1.10 (3H, s, CH_3_), 0.97 (3H, s, CH_3_), 0.84 (3H, s, CH_3_). ^13^C-NMR (CDCl_3_, 100 MHz) δ: 181.03, 155.89, 135.10, 133.27, 129.44, 128.80, 52.27, 47.35, 43.18, 40.14, 35.35, 32.24, 26.71, 19.25, 18.50, 11.22. IR (KBr) v, cm^−1^: 1650 (C=N). HRMS (ESI, +H): Theoretical mass calculated for [C_17_H_21_ClN_2_ + H]: 289.1472; Found: 289.1456. 

*(1E,2E)-1-(4-Bromobenzylidene)-2-(1,7,7-trimethylbicyclo[2.2.1]heptan-2-ylidene)hydrazine* (**3d**). Purified by column chromatography on silica gel (70–230 mesh, AcOEt/*n*-Hex 10%). Yield: 57%, mp: 60–62 °C. ^1^H-NMR (CDCl_3_, 400 MHz) δ: 8.28 (1H, s, CH=N), 7.50–7.65 (4H, m, phenyl), 2.15–2.70 (2H, m, N=C-CH_2_), 1.94 (1H, t, CH, *J* = 4.28 Hz), 1.20–1.90 (4H, m, CH_2_-CH_2_), 1.09 (3H, s, CH_3_), 0.97 (3H, s, CH_3_), 0.84 (3H, s, CH_3_). ^13^C-NMR (CDCl_3_, 100 MHz) δ: 183.38, 156.61, 133.91, 132.09, 129.67, 125.01, 52.97, 48.17, 44.11, 36.26, 32.85, 27.51, 19.86, 19.02, 11.47. IR (KBr) v, cm^−1^: 1650, (C=N). HRMS (ESI, +H): Theoretical mass calculated for [C_17_H_21_BrN_2_ + H]: 333.0966; Found: 333.0957.

*4-((E)-((E)-(1,7,7-Trimethylbicyclo[2.2.1]heptan-2-ylidene)hydrazono)methyl)phenol* (**3e**). Purified by column chromatography on silica gel (70–230 mesh, AcOEt/*n*-Hex 20%). Yield: 70%, mp: 157–160 °C. ^1^H-NMR (DMSO, 400 MHz) δ: 9.95 (1H, s, OH), 8.24 (1H, s, CH=N), 6.75–7.65 (4H, m, phenyl), 2.05–2.60 (2H, m, N=C-CH_2_), 1.90 (1H, t, CH, *J* = 4.20 Hz), 1.20–1.80 (4H, m, CH_2_-CH_2_), 0.99 (3H, s, CH_3_), 0.92 (3H, s, CH_3_), 0.76 (3H, s, CH_3_). ^13^C-NMR (DMSO, 100 MHz) δ: 179.88, 159.74, 157.18, 129.61, 125.39, 115.45, 52.01, 47.21, 43.13, 35.33, 32.26, 26.72, 19.22, 18.47, 11.28. IR (KBr) v, cm^−1^: 1655 (C=N). HRMS (ESI, +H): Theoretical mass calculated for [C_17_H_22_N_2_O + H]: 271.1810; Found: 271.1797.

*(1E,2E)-1-(4-Methoxybenzylidene)-2-(1,7,7-trimethylbicyclo[2.2.1]heptan-2-ylidene)hydrazine* (**3f**). Purified by column chromatography on silica gel (70–230 mesh, AcOEt/*n*-Hex 20%). Yield: 75%, mp: 67–69 °C. ^1^H-NMR (DMSO, 400 MHz) δ: 8.29 (1H, s, CH=N), 6.95–7.75 (4H, m, phenyl), 2.00–2.55 (2H, m, N=C-CH_2_), 1.91 (1H, t, CH, *J* = 4.20 Hz), 1.20–1.80 (4H, m, CH_2_-CH_2_), 1.00 (3H, s, CH_3_), 0.93 (3H, s, CH_3_), 0.76 (3H, s, CH_3_). ^13^C-NMR (DMSO, 100 MHz) δ: 179.88, 159.74, 157.18, 129.61, 125.39, 115.45, 52.01, 47.21, 43.13, 35.33, 32.26, 26.72, 19.22, 18.47, 11.28. IR (KBr) v, cm^−1^: 1575 (C=N). HRMS (ESI, +H): Theoretical mass calculated for [C_18_H_24_N_2_O + H]: 285.1967; Found: 285.1952.

*(1E,2E)-1-(4-Nitrobenzylidene)-2-(1,7,7-trimethylbicyclo[2.2.1]heptan-2-ylidene)hydrazine* (**3g**). Purified by filtration and washing with cold ethyl ether. Yield: 36%, mp: 100–102 °C. ^1^H-NMR (DMSO, 400 MHz) δ: 8.48 (1H, s, CH=N), 8.00–8.35 (4H, m, phenyl), 2.05–2.65 (2H, m, N=C-CH_2_), 1.94 (1H, t, CH, *J* =3.20 Hz), 1.20–1.90 (4H, m, CH_2_-CH_2_), 1.02 (3H, s, CH_3_), 0.94 (3H, s, CH_3_), 0.78 (3H, s, CH_3_). ^13^C-NMR (DMSO, 100 MHz) δ: 181.89, 155.20, 148.37, 140.42, 128.86, 123.98, 52.52, 47.53, 43.20, 35.43, 32.25, 26.72, 19.32, 18.55, 11.26. IR (KBr), v, cm^−1^: 1612, 1651 (C=N). HRMS (ESI, +H): Theoretical mass calculated for [C_17_H_21_N_3_O_2_ + H]: 300.1712; Found: 300.1710.

*(1E,2E)-1-(3-Chlorobenzylidene)-2-(1,7,7-trimethylbicyclo[2.2.1]heptan-2-ylidene)hydrazine* (**3h**). Purified by column chromatography on silica gel (70–230 mesh, AcOEt/*n*-Hex 10%). Yield: 70%, mp: 58–60 °C. ^1^H-NMR (DMSO, 400 MHz) δ: 8.33 (1H, s, CH=N), 7.48–7.82 (4H, m, phenyl), 2.09–2.61 (2H, m, N=C-CH_2_), 1.92 (1H, t, CH, *J* = 4.04 Hz), 1.22–1.84 (4H, m, CH_2_-CH_2_), 1.00 (3H, s, CH_3_), 0.93 (3H, s, CH_3_), 0.80 (3H, s, CH_3_). ^13^C-NMR (DMSO, 100 MHz) δ: 181.34, 155.66, 136.57, 133.51, 130.65, 130.27, 127.18, 126.47, 52.35, 47.43, 35.39, 32.25, 26.72, 19.29, 18.53, 11.27. IR (KBr) v, cm^−1^: 1709, 1655 (C=N). HRMS (ESI, +H): Theoretical mass calculated for [C_17_H_21_ClN_2_ + H]: 289.1472; Found: 289.1477.

*3-((E)-((E)-(1,7,7-Trimethylbicyclo[2.2.1]heptan-2-ylidene)hydrazono)methyl)benzonitrile* (**3i**). Purified by column chromatography on silica gel (70–230 mesh, AcOEt/*n*-Hex 10%). Yield: 85%, mp: 93–94 °C. ^1^H-NMR (DMSO, 400 MHz) δ: 8.39 (1H, s, CH=N), 7.65–8.18 (4H, m, phenyl), 2.49–2.52 (2H, m, N=C-CH_2_), 2.10 (1H, t, CH, *J* = 3.92 Hz), 1.03–2.00 (4H, m, CH_2_-CH_2_), 1.01 (3H, s, CH_3_), 0.94 (3H, s, CH_3_), 0.77 (3H, s, CH_3_). ^13^C-NMR (DMSO, 100 MHz) δ: 181.59, 155.05, 135.52, 133.74, 131.33, 129.95, 118.27, 111.83, 52.31, 47.37, 43.09, 35.33, 32.15, 26.63, 19.20, 18.44, 11.16. IR (KBr) v, cm^−1^: 1647 (C=N). HRMS (ESI, +H): Theoretical mass calculated for [C_18_H_21_N_3_ + H]: 280.1814; Found: 280.1822.

*(1E,2E)-1-(3-Methoxybenzylidene)-2-(1,7,7-trimethylbicyclo[2.2.1]heptan-2-ylidene)hydrazine* (**3j**). Purified by column chromatography on silica gel (70–230 mesh, AcOEt/*n*-Hex 10%). Yield: 61%, oil. ^1^H-NMR (DMSO, 400 MHz) δ: 8.29 (1H, s, CH=N), 7.00–7.37 (4H, m, phenyl), 3.78 (3H, s, -OCH_3_), 2.09–2.51 (2H, m, N=C-CH_2_), 1.91 (1H, t, CH, *J* = 4.04 Hz), 1.25–1.38 (4H, m, CH_2_-CH_2_), 1.00 (3H, s, CH_3_), 0.93 (3H, s, CH_3_), 0.77 (3H, s, CH_3_). ^13^C-NMR (DMSO, 100 MHz) δ: 182.46, 159.77, 157.26, 136.12, 129.60, 121.31, 116.87, 111.87, 55.31, 52.66, 47.92, 43.89, 35.97, 32.63, 27.29, 19.63, 18.81, 11.28. IR (KBr) v, cm^−1^: 1653 (C=N). HRMS (ESI, +H): Theoretical mass calculated for [C_18_H_24_N_2_O + H]: 285.1967; Found: 285.1964. 

*(1E,2E)-1-(3-Nitrobenzylidene)-2-(1,7,7-trimethylbicyclo[2.2.1]heptan-2-ylidene)hydrazine* (**3k**). Purified by filtration and washing with cold ethyl ether. Yield: 50%, mp: 110–113 °C. ^1^H-NMR (DMSO, 400 MHz) δ: 8.59 (1H, s, phenyl), 8.49 (1H, s, CH=N), 7.70–8.35 (3H, m, phenyl), 2.10–2.65 (2H, m, N=C-CH_2_), 1.94 (1H, t, CH, *J* = 4.08 Hz), 1.20–1.85 (4H, m, CH_2_-CH_2_), 1.02 (3H, s, CH_3_), 0.94 (3H, s, CH_3_), 0.78 (3H, s, CH_3_). ^13^C-NMR (DMSO, 100MHz) δ: 184.41, 155.04, 148.66, 136.58, 133.66, 129.64, 124.80, 122.55, 52.97, 48.07, 43.88, 36.19, 32.63, 27.28, 19.64, 18.80, 11.22; IR (KBr) v, cm^−1^: 1648 (C=N); HRMS (ESI, +H): Theoretical mass calculated for C_17_H_23_N_3_O_2_ + H: 300.1712; Found: 300.1705. 

*(1E,2E)-1-(2-Fluorobenzylidene)-2-(1,7,7-trimethylbicyclo[2.2.1]heptan-2-ylidene)hydrazine* (**3l**). Purified by column chromatography on silica gel (70–230 mesh, AcOEt/*n*-Hex 5%). Yield: 68%, oil. ^1^H-NMR (DMSO, 400 MHz) δ: 8.44 (1H, s, CH=N), 7.25–7.90 (4H, m, phenyl), 2.10–2.65 (2H, m, N=C-CH_2_), 1.92 (1H, t, CH, *J* = 4.15 Hz), 1.20–1.85 (4H, m, CH_2_-CH_2_), 1.01 (3H, s, CH_3_), 0.94 (3H, s, CH_3_), 0.78 (3H, s, CH_3_). ^13^C-NMR (DMSO,100 MHz) δ: 181.44, 162.29, 159.79, 149.88, 149.85, 132.67, 132.58, 127.41, 127.39, 124.72, 124.69, 121.68, 121.58, 116.09, 115.88, 52.25, 47.35, 43.07, 35.32, 32.13, 26.62, 19.20, 18.44, 11.15. IR (KBr) v, cm^−1^: 1640 (C=N). MS (ESI, +H): 278. 

*(1E,2E)-1-(2-Chlorobenzylidene)-2-(1,7,7-trimethylbicyclo[2.2.1]heptan-2-ylidene)hydrazine* (**3m**). Purified by column chromatography on silica gel (70–230 mesh, AcOEt/*n*-Hex 5%). Yield: 51%, mp: 66–69 °C. ^1^H-NMR (DMSO, 400 MHz) δ: 8.58 (1H, s, CH=N), 7.40–8.05 (3H, m, phenyl), 2.05–2.65 (2H, m, N=C-CH_2_), 1.93 (1H, t, CH, *J* = 4.04 Hz), 1.20–1.85 (4H, m, CH_2_-CH_2_), 1.01 (3H, s, CH_3_), 0.94 (3H, s, CH_3_), 0.78 (3H, s, CH_3_). ^13^C-NMR (DMSO, 100 MHz) δ: 181.93, 152.98, 133.80, 132.10, 131.20, 129.86, 127.79, 127.44, 52.31, 47.38, 43.08, 35.38, 32.12, 26.62, 19.20, 18.44, 11.15. IR (KBr) v, cm^−1^: 1651 (C=N); HRMS (ESI, +H): Theoretical mass calculated for [C_17_H_21_ClN_2_ + H]: 289.1472; Found: 289.1471. 

*(1E,2E)-1-(2-Bromobenzylidene)-2-(1,7,7-trimethylbicyclo[2.2.1]heptan-2-ylidene)hydrazine* (**3n**). Purified by column chromatography on silica gel (70–230 mesh, AcOEt/*n*-Hex 5%). Yield: 55%, mp: 88–90 °C. ^1^H-NMR (DMSO, 400 MHz) δ: 8.52 (1H, s, CH=N), 7.39–8.00 (4H, m, phenyl), 1.94–2.51 (2H, m, N=C-CH_2_), 1.93 (1H, t, CH, *J* = 4.02 Hz), 1.23–1.80 (4H, m, CH_2_-CH_2_), 1.02 (3H, s, CH_3_), 0.94 (3H, s, CH_3_), 0.78 (3H, s, CH_3_). ^13^C-NMR (DMSO, 100 MHz) δ: 181.99, 155.28, 133.08, 132.64, 132.34, 128.15, 127.95, 124.12, 52.32, 47.38, 43.07, 43.07, 35.40, 32.11, 26.61, 19.20, 18.43, 11.15. IR (KBr) v, cm^−1^: 1657 (C=N). HRMS (ESI, +H): Theoretical mass calculated for [C_17_H_21_BrN_2_ + H]: 333.0966; Found: 333.0978.

*2-((E)-((E)-(1,7,7-Trimethylbicyclo[2.2.1]heptan-2-ylidene)hydrazono)methyl)phenol* (**3o**). Purified by column chromatography on silica gel (70–230 mesh, AcOEt/*n*-Hex 10%). Yield: 64%, mp: 54–57 °C. ^1^H-NMR (CDCl_3_, 400 MHz) δ: 12.00 (1H, s, OH), 8.61 (1H, s, CH=N), 6.85–7.40 (4H, m, phenyl), 2.10–2.75 (2H, m, N=C-CH_2_), 2.00 (1H, t, CH, *J* =4.28 Hz), 1.20-1.95 (4H, m, CH_2_-CH_2_), 1.12 (3H, s, CH_3_), 0.98 (3H, s, CH_3_), 0.83 (3H, s, CH_3_). ^13^C-NMR (CDCl_3_, 100 MHz) δ: 185.26, 162.27, 160.05, 132.48, 132.12, 119.46, 118.18, 117.00, 53.23, 48.44, 44.02, 36.49, 32.78, 27.43, 19.82, 18.96, 11.40. IR (KBr) v, cm^−1^: 1650, 1624 (C=N). HRMS (ESI, +H): Theoretical mass calculated for [C_17_H_22_N_2_O + H]: 271.1810; Found: 271.1797. 

*(1E,2E)-1-(2-Nitrobenzylidene)-2-(1,7,7-trimethylbicyclo[2.2.1]heptan-2-ylidene)hydrazine* (**3p**). Purified by column chromatography on silica gel (70–230 mesh, AcOEt/*n*-Hex 10%). Yield: 77%, mp: 72–75 °C. ^1^H-NMR (CDCl_3_, 400 MHz) δ: 8.53 (1H, s, CH=N), 7.65–8.05 (4H, m, phenyl), 2.05–2.55 (2H, m, N=C-CH_2_), 1.93 (1H, t, CH, *J* = 4.28 Hz), 1.20–1.95 (4H, m, CH_2_-CH_2_-), 1.01 (3H, s, CH_3_), 0.94 (3H, s, CH_3_), 0.78 (3H, s, CH_3_). ^13^C-NMR (CDCl_3_, 100 MHz) δ: 181.48, 152.76, 148.73, 133.36, 131.13, 129.38, 128.34, 124.32, 52.30, 47.43, 43.04, 35.31, 32.08, 26.64, 19.18, 18.42, 11.14. IR (KBr) v, cm^−1^: 1647 (C=N). HRMS (ESI, +H): Theoretical mass calculated for [C_17_H_21_N_3_O_2_ + H]: 300.1712; Found: 300.1707. 

*(1E,2E)-1-(2,3-Dichlorobenzylidene)-2-(1,7,7-trimethylbicyclo[2.2.1]heptan-2-ylidene)hydrazine* (**3q**). Purified by column chromatography on silica gel (70–230 mesh, AcOEt/*n*-Hex 10%). Yield: 78%, mp: 69–72 °C. ^1^H-NMR (DMSO, 400 MHz) δ: 8.60 (1H, s, CH=N), 7.42–8.00 (3H, m, phenyl), 2.10–2.60 (2H, m, N=C-CH_2_-), 1.92 (1H, t, CH, *J* = 4.24 Hz), 1.03–1.70 (4H, m, CH_2_-CH_2_), 1.02 (3H, s, CH_3_), 0.94 (3H, s, CH_3_), 0.78 (3H, s, CH_3_). ^13^C-NMR (DMSO, 100 MHz) δ: 182.43, 153.04, 133.74, 132.39, 132.28, 131.75, 128.43, 126.55, 52.49, 47.52, 43.17, 35.49, 32.20, 26.70, 19.30, 18.53, 11.23. IR (KBr) v, cm^−1^: 1650 (C=N). HRMS (ESI, +H): Theoretical mass calculated for [C_17_H_20_Cl_2_N_2_ + H]: 323.1082; Found: 323.1082.

*3-((E)-((E)-(1,7,7-Trimethylbicyclo[2.2.1]heptan-2-ylidene)hydrazono)methyl)benzene-1,2-diol* (**3r**). Purified by column chromatography on silica gel (70–230 mesh, AcOEt/*n*-Hex 10%). Yield: 73%, mp: 104–106 °C. ^1^H-NMR (CDCl_3_, 400 MHz) δ: 12.36 (1H, s, OH), 8.59 (1H, s, CH=N), 6.75–7.01 (3H, m, phenyl), 2.17–2.75 (2H, m, N=C-CH_2_), 2.01 (1H, t, CH, *J* = 4.28 Hz), 1.25–1.95 (4H, m, CH_2_-CH_2_), 1.12 (3H, s, CH_3_), 0.98 (3H, s, CH_3_), 0.83 (3H, s, CH_3_). ^13^C-NMR (CDCl_3_, 100 MHz) δ: 185.29, 162.20, 147.08, 144.95, 122.99, 119.77, 117.84, 117.39, 53.30, 48.47, 44.04, 36.43, 32.76, 27.42, 19.83, 18.95, 11.38. IR (KBr) v, cm^−1^: 1643 (C=N). HRMS (ESI, +H): Theoretical mass calculated for [C_17_H_22_N_2_O_2_ + H]: 287.1760; Found: 287.1785.

*4-((E)-((E)-(1,7,7-Trimethylbicyclo[2.2.1]heptan-2-ylidene)hydrazono)methyl)benzene-1,3-diol* (**3s**). Purified by filtration and washing with cold ethyl ether. Yield: 25%, mp: 183–185 °C. ^1^H-NMR (DMSO, 500 MHz) δ: 12.01 (1H, s, OH), 10.15 (1H, s, OH), 8.59 (1H, s, CH=N), 6.25–7.35 (3H, m, phenyl), 2.08–2.65 (2H, m, N=C-CH_2_), 1.94 (1H, t, CH, *J* = 4.00 Hz), 1.30–1.90 (4H, m, CH_2_-CH_2_), 1.00 (3H, s, CH_3_), 0.94 (3H, s, CH_3_), 0.74 (3H, s, CH_3_). ^13^C-NMR (DMSO, 120 MHz) δ: 181.71, 161.66, 161.63, 161.12, 110.22, 107.84, 102.33, 52.39, 47.62, 43.18, 35.41, 32.25, 26.68, 19.28, 18.51, 11.26. IR (KBr) v, cm^−1^: 1661 (C=N). HRMS (ESI, +H): Theoretical mass calculated for [C_17_H_22_N_2_O_2_ + H]: 287.1760; Found: 287.1756.

*4-((E)-((E)-(1,7,7-Trimethylbicyclo[2.2.1]heptan-2-ylidene)hydrazono)methyl)benzene-1,2-diol* (**3t**). Purified by filtration and washing with cold ethyl ether. Yield: 38%, mp: 186–190 °C. ^1^H-NMR (DMSO, 400 MHz) δ: 8.16 (1H, s, OH), 7.24 (1H, s, OH), 8.16 (1H, s, CH=N), 6.76–7.24 (3H, m, phenyl), 2.08–2.15 (2H, m, N=C-CH_2_), 1.91 (1H, t, CH, *J* = 4.04 Hz), 1.21–1.85 (4H, m, CH_2_-CH_2_), 0.99 (3H, s, CH_3_), 0.92 (3H, s, CH_3_), 0.76 (3H, s, CH_3_). ^13^C-NMR (DMSO, 100 MHz) δ: 157.57, 146.84, 143.69, 127.53, 122.90, 115.17, 113.92, 52.90, 48.11, 43.84, 36.14, 32.64, 27.24, 19.64, 18.78, 11.29. IR (KBr) v, cm^−1^: 1663 (C=N). HRMS (ESI, +H): Theoretical mass calculated for [C_17_H_22_N_2_O_2_ + H]: 287.1760; Found: 287.1778.

*2-((E)-((E)-(1,7,7-Trimethylbicyclo[2.2.1]heptan-2-ylidene)hydrazono)methyl)benzene-1,4-diol* (**3u**). Purified by filtration and washing with cold ethyl ether. Yield: 64%, mp: 218–220 °C. ^1^H-NMR (DMSO, 400 MHz) δ: 10.94 (1H, s, OH), 9.00 (1H, s, OH), 8.60 (1H, s, CH=N), 6.74–6.94 (3H, m, phenyl), 2.09–2.60 (2H, m, N=C-CH_2_), 1.96 (1H, t, CH, *J* = 3.92 Hz), 1.24–1.87 (4H, m, CH_2_-CH_2_), 1.02 (3H, s, CH_3_), 0.94 (3H, s, CH_3_), 0.76 (3H, s, -CH_3_). ^13^C-NMR (DMSO, 100 MHz) δ: 185.44, 161.54, 153.99, 148.00, 120.10, 117.89, 117.55, 117.16, 53.06, 48.24, 43.78, 36.30, 32.55, 27.20, 19.61, 18.74, 11.18. IR (KBr) v, cm^−1^: 1651 (C=N). HRMS (ESI, +H): Theoretical mass calculated for [C_17_H_22_N_2_O_2_ + H]: 287.1760; Found: 287.1773.

*4-Methoxy-2-((E)-((E)-(1,7,7-trimethylbicyclo[2.2.1]heptan-2-ylidene)hydrazono)methyl)phenol* (**3v**). Purified by column chromatography on silica gel (70–230 mesh, AcOEt/*n*-Hex 10%). Yield: 78%, mp: 77–80 °C. ^1^H-NMR (DMSO, 400 MHz) δ: 12.07 (1H, 1s, OH), 8.64 (1H, s, CH=N), 6.47–7.47 (3H, m, phenyl), 3.78 (3H, s, OCH_3_), 2.00–2.70 (2H, m, N=C-CH_2_), 1.96 (1H, t, CH, *J* = 4.04 Hz), 1.23–1.87 (4H, m, CH_2_-CH_2_), 1.01 (3H, s, CH_3_), 0.93 (3H, s, CH_3_), 0.76 (3H, s, CH_3_). ^13^C-NMR (DMSO, 100 MHz) δ: 182.32, 163.37, 162.91, 162.06, 161.46, 161.04, 160.66, 133.31, 132.70, 111.40, 111.37, 107.10, 106.68, 100.95, 100.83. IR (KBr) v, cm^−1^: 1651, 1633 (C=N). HRMS (ESI, +H): Theoretical mass calculated for [C_18_H_24_N_2_O_2_ + H]: 301.1916; Found: 301.1915.

*5-Nitro-2-((E)-((E)-(1,7,7-trimethylbicyclo[2.2.1]heptan-2-ylidene)hydrazono)methyl)phenol* (**3w**). Purified by filtration and washing with cold ethanol. Yield: 54%, mp: 138–141 °C. ^1^H-NMR (DMSO, 400 MHz) δ: 12.77 (1H, s, OH), 8.80 (1H, s, CH=N), 7.12–8.61 (3H, m, phenyl), 2.14–2.65 (2H, m, N=C-CH_2_), 1.98 (1H, t, CH, *J* = 3.76 Hz), 1.41–1.97 (4H, m, CH_2_-CH_2_), 1.38 (3H, s, CH_3_), 1.36 (3H, s, CH_3_), 1.35 (3H, s, CH_3_). ^13^C-NMR (DMSO, 100 MHz) δ: 184.41, 164.08, 158.45, 139.80, 127.51, 126.75, 118.49, 117.34, 52.73, 47.70, 43.15, 35.44, 32.14, 26.54, 19.25, 18.46, 11.10. IR (KBr) v, cm^−1^: 1628 (C=N). HRMS (ES, +H): Theoretical mass calculated for [C_17_H_21_N_3_O_3_ + H]: 316.1661; Found: 316.1651.

*2-((E)-((E)-(1,7,7-Trimethylbicyclo[2.2.1]heptan-2-ylidene)hydrazono)methyl)pyridine* (**3x**). Purified by column chromatography on silica gel (70–230 mesh, AcOEt/*n*-Hex 30%). Yield: 38%, oil. ^1^H-NMR (DMSO, 400 MHz) δ: 8.64 (1H, m, phenyl), 8.19 (1H, s, CH=N), 7.80–8.00 (3H, m, phenyl), 2.05–2.55 (2H, m, N=C-CH_2_), 1.92 (1H, t, CH, *J* = 4.12 Hz), 1.05–1.97 (4H, m, CH_2_-CH_2_), 1.01 (3H, s, CH_3_), 0.93 (3H, s, CH_3_), 0.77 (3H, s, CH_3_). ^13^C-NMR (DMSO, 100 MHz) δ: 181.05, 156.64, 152.95, 149.58, 136.79, 124.91, 120.86, 52.37, 47.45, 43.18, 35.33, 32.20, 26.70, 19.25, 18.51, 11.20. IR (KBr) v, cm^−1^: 1641 (C=N). HRMS (ESI, +H): Theoretical mass calculated for [C_16_H_21_N_3_ + H]: 256.1814; Found: 256.1800.

*1,7,7-Trimethylbicyclo[2.2.1]heptan-2-amine hydrochloride* **5** [26]. A stirred solution of the oxime **4** (2.0 g, 12.40 mmol) in MeOH (120 mL) was hydrogenated using 5 bar pressure of H_2_ and 10% Pd/C (0.12 g) for 48 h at 80 °C. The reaction mixture was filtered, rotary evaporated, and the oily residue dissolved in acetone (20 mL). The solution was acidified (pH = 2) with gaseous HCl. The precipitate of the hydrochloride **5** was collected, washed with cold acetone, and dried under vacuum. Yield: 0.75 g, 50%. ^1^H-NMR (DMSO, 400 MHz) δ: 7.96 (3H, sbr, NH_3_), 3.00 (1H, m, CH-NH_3_), 1.70–1.89 (3H, m, N=C-CH_2_, CH), 1.10–1.69 (4H, m, CH_2_-CH_2_), 0.94 (3H, s, CH_3_), 0.90 (3H, s, CH_3_), 0.81 (3H, s, CH_3_). ^13^C-NMR (DMSO, 100 MHz) δ: 57.17, 47.48, 46.66, 44.15, 35.75, 35.71, 26.17, 20.02, 19.93, 11.42. IR (KBr) v, cm^−1^: 3039 (NH). MS (*m*/*z*, %): 153 (M^+^, 43), 108 (25), 95 (99), 82 (100), 70 (24).

### 3.5. General Synthetic Procedure for the Imine Derivatives ***7***

The appropriate amine (1.5–2 equivalents) was added with stirring to a solution of nitroimine **1** (1.0–3.0 mmol) in CHCl_3_ or CH_3_CN (15–20 mL). The reaction mixture was refluxed until TLC indicated the consumption of **1** or lack of further reaction (3–72 h). The products **7a**–**7d** were obtained pure after adequate procedure: the reaction solution (in this case chloroform) was extracted with a saturated aqueous solution of NaHCO_3_ (3 × 5 mL). The organic layer was washed with a saturated aqueous solution of brine (5 mL) and dried over MgSO_4_. The solvents were removed by rotary evaporation to furnish the product pure in accordance with spectra analyses (in these reactions the use of recrystallized oxime was necessary). 

The products **7e**–**7n**, after evaporation of the solvent, were purified by column chromatography on silica gel (70–230 mesh, AcEOt/*n*-Hex 10%–50%).

*(E)-1-Phenyl-N-(1,7,7-trimethylbicyclo[2.2.1]heptan-2-ylidene)methanamine* (**7a**) [27]. Yield: 55%, oil. ^1^H-NMR (MeOD, 400 MHz) δ 7.21–7.35 (5H, m, phenyl), 4.47 (2H, dd, *J* =14.3 Hz), 1.95–2.50 (3H, m, N=C-CH_2_, CH), 1.10–1.70 (4H, m, CH_2_-CH_2_), 1.00 (3H, s, CH_3_), 0.96 (3H, s, CH_3_), 0.79 (3H, s, CH_3_). ^13^C-NMR (MeOD, 100 MHz) δ: 57.17, 47.48, 46.66, 44.15, 35.75, 35.71, 26.17, 20.02, 19.93, 11.42. IR (KBr) v, cm^−1^: 3039. MS (ESI, +H): 242.

*(E)-N-(1,7,7-Trimethylbicyclo[2.2.1]heptan-2-ylidene)butan-1-amine* (**7b**) [28]. Yield: 70%, oil. ^1^H-NMR (MeOD, 400 MHz) δ: 3.15–3.27 (m, 2H, N-CH_2_), 1.15–2.40 (11H, m, N=C-CH_2_, CH, CH_2_-CH_2_-camphor, CH_2_-CH_2_-side chain), 0.96 (s, 3H, CH_3_), 0.93 (3H, s, CH_3_ side chain), 0.92 (3H, s, CH_3_), 0.75 (s, 3H, CH_3_). ^13^C-NMR (MeOD, 100 MHz) δ: 53.67, 52.34, 47.09, 44.08, 35.62, 32.94, 32.45, 27.75, 20.93, 19.79, 19.22, 14.28, 11.71. IR (KBr) v, cm^−1^: 1677 (C=N). MS (ESI, +H): 208. 

*(E)-N-(1,7,7-Trimethylbicyclo[2.2.1]heptan-2-ylidene)cyclopropanamine* (**7c**). Yield: 65%, oil. ^1^H-NMR (MeOD, 400 MHz) δ: 2.65–2.75 (m, 1H, N-CH), 1.95–2.50 (2H, m, N=C-CH_2_) 1.93 (t, 1H, CH, *J* = 7.2 Hz), 1.15–1.90 (4H, m, CH_2_-CH_2_), 0.93 (3H, s, CH_3_), 0.91 (3H, s, CH_3_), 0.76 (3H, s, CH_3_), 0.71–0.74 (m, 4H, cyclopropyl). ^13^C-NMR (MeOD, 100 MHz) δ: 52.82, 46.45, 43.31, 35.34, 33.52, 31.90, 26.99, 19.25, 18.75, 11.44, 7.61, 7.38. IR (KBr) v, cm^−1^: 1677 (C=N). HRMS (ESI, +H): Theoretical mass calculated for C_13_H_21_N + H: 192.1752; Found: 192.1744.

*(E)-N-(1,7,7-Trimethylbicyclo[2.2.1]heptan-2-ylidene)cyclohexanamine* (**7d**) [29]. Yield: 73%, oil. ^1^H-NMR (MeOD, 400 MHz) δ: 3.07 (m, 1H, N-CH), 1.15–2.55 (17H, m, N=C-CH_2_, CH, CH_2_-CH_2_-camphor, CH_2_(CH_2_)_3_CH_2_-cyclohexyl), 0.96 (3H, s, -CH_3_), 0.91 (3H, s, -CH_3_), 0.75 (3H, s, -CH_3_). ^13^C-NMR (MeOD, 100 MHz) δ: 52.82, 46.45, 43.31, 35.34, 33.52, 31.90, 26.99, 19.25, 18.75, 11.44, 7.61, 7.38. IR (KBr) v, cm^−1^: 1683 (C=N). GC: 100%, MS (*m*/*z*, %): 233 (M^+^, 25), 205 (100), 136 (17), 109 (16), 95 (16).

*(E)-4-Fluoro-N-(1,7,7-trimethylbicyclo[2.2.1]heptan-2-ylidene)aniline* (**7e**). Yield: 79%, oil. ^1^H-NMR (MeOD, 400 MHz) δ: 7.07 (4H, m, phenyl), 2.15–2.25 (1H, m, -=C-CH_a_-, 1.75–1.97 (4H, m, -N=C-CH_b_, CH, CH_2_), 1.25–1.55 (2H, m, CH_2_), 1.07 (3H, s, CH_3_), 1.00 (3H, s, CH_3_), 0.87 (3H, s, CH_3_). ^13^C-NMR (MeOD, 100 MHz) δ: 189.73, 162.38, 159.99, 148.78, 148.76, 122.57, 122.49, 116.97, 116.75, 55.56, 45.30, 37.60, 33.21, 28.25, 20.06, 19.44, 11.71. IR (KBr) v, cm^−1^: 1682 (C=N). HRMS (ESI, +H): Theoretical mass calculated for [C_16_H_20_FN + H]: 246.1658; Found: 246.1658.

*(E)-4-Chloro-N-(1,7,7-trimethylbicyclo[2.2.1]heptan-2-ylidene)aniline* (**7f**). Yield: 52%, mp: 34–36 °C. ^1^H-NMR (MeOD, 400 MHz) δ: 6.60–7.27 (4H, m, phenyl); 2.14–2.22 (1H, m, -=N=C-CH_a_); 1.68–1.95 (4H, m, N=C-CH_b_, CH, CH_2_); 1.15–1.55 (2H, m, CH_2_); 1.07 (3H, s, CH_3_); 0.97 (3H, s, CH_3_); 0.85 (3H, s, CH_3_). ^13^C-NMR (MeOD, 100 MHz) δ: 185.74, 150.77, 128.76, 128.28, 120.85, 54.09, 47.20, 43.74, 36.23, 31.99, 27.38, 19.54, 18.99, 11.16. IR (KBr) v, cm^−1^: 1680 (C=N). HRMS (ESI, +H): Theoretical mass calculated for [C_16_H_20_ClN + H]: 262.1636; Found: 262.1635.

*(E)-B-Bromo-N-(1,7,7-trimethylbicyclo[2.2.1]heptan-2-ylidene)aniline* (**7g**). Yield: 73%, oil. ^1^H-NMR (MeOD, 400 MHz) δ: 6.61–7.39 (4H, m, phenyl), 2.10–2.20 (1H, m, N=C-CH_a_), 1.65–1.95 (4H, m, N=C-CH_b_, CH, -CH_2_-), 1.20–1.55 (2H, m, CH_2_), 1.07 (3H, s, CH_3_), 0.97 (3H, s, CH_3_), 0.85 (3H, s, CH_3_). ^13^C-NMR (MeOD, 100 MHz) δ: 184.22, 151.10, 131.73, 121.53, 114.96, 53.79, 46.73, 43.16, 35.48, 31.56, 26.77, 19.25, 18.77, 11.23. IR (KBr) v, cm^−1^: 1604 (C=N). HRMS (ESI, +H): Theoretical mass calculated for [C_16_H_20_BrN + H]: 306.0857; Found: 306.0856.

*(E)-4-Hydroxy-N-(1,7,7-trimethylbicyclo[2.2.1]heptan-2-ylidene)aniline* (**7h**) [30]. Yield: 81%, mp: 142–144 °C. ^1^H-NMR (DMSO, 400 MHz) δ: 9.02 (1H, s, OH); 6.50–6.60 (4H, m, phenyl), 2.11–2.20 (1H, m, N=C-CH_a_), 1.70–1.90 (4H, m, N=C-CH_b_, CH, CH_2_), 1.20–1.45 (2H, m, CH_2_), 0.96 (3H, s, CH_3_), 0.92 (3H, s, CH_3_), 0.77 (3H, s, CH_3_). ^13^C-NMR (DMSO, 100 MHz) δ: 182.32, 153.14, 143.17, 120.47, 115.39, 53.56, 46.48, 43.24, 40.13, 35,64, 31.70, 26.90, 19.28, 18.82, 11.46. IR (KBr) v, cm^−1^: 1665 (C=N). GC: 100%, MS (*m*/*z*, %): 243 (M^+^, 100), 160 (40), 135 (46), 134 (20), 95 (32).

*(E)-4-Methoxy-N-(1,7,7-trimethylbicyclo[2.2.1]heptan-2-ylidene)aniline* (**7i**) [31]. Yield: 90%, mp: 41–43 °C. ^1^H-NMR (MeOD, 400 MHz) δ: 6.60–6.90 (4H, m, phenyl); 3.75 (3H, s, OCH_3_), 2.15–2.26 (1H, m, N=C-CH_a_), 1.75–1.95 (4H, m, N=C-CH_b_, CH, CH_2_), 1.25–1.55 (2H, m, CH_2_), 0.96 (3H, s, CH_3_), 0.92 (3H, s, CH_3_), 0.77 (3H, s, CH_3_). ^13^C-NMR (MeOD, 100 MHz) δ: 189.25, 158.06, 145.45, 122.13, 115.63, 56.01, 55.47, 45.32, 37.71, 33.26, 28.30, 19.45, 11.78. IR (KBr) v, cm^−1^: 1675 (C=N). GC: 99%, MS (*m*/*z*, %): 257 (M^+^, 100%), 174 (30), 149 (33), 95 (23).

*(E)-2-Hydroxy-N-(1,7,7-trimethylbicyclo[2.2.1]heptan-2-ylidene)aniline* (**7j**) [32]. Yield: 53%, mp: 94–96 °C. ^1^H-NMR (DMSO, 400 MHz) δ: 8.83(1H, s, OH); 6.50–6.85 (4H, m, phenyl), 2.10–2.25 (1H, m, N=C-CH_a_), 1.65–1.90 (4H, m, N=C-CH_b_, CH, CH_2_), 1.10–1.25 (2H, m, CH_2_), 0.99 (3H, s, CH_3_), 0.92 (3H, s, CH_3_), 0.83 (3H, s, CH_3_). ^13^C-NMR (DMSO, 100 MHz) δ: 183.86, 146.58, 139.20, 123.47, 120.37, 119.18, 115.75, 53.75, 46.98, 43.24, 36.58, 31.76, 26.96, 19.39, 18.89, 11.34. IR (KBr) v, cm^−1^: 1668 (C=N). GC: 96%, MS (*m*/*z*, %): 243 (88), 228 (60), 172 (62), 134 (68), 95 (100).

*(E)-2,5-Dimethoxy-N-(1,7,7-trimethylbicyclo[2.2.1]heptan-2-ylidene)aniline* (**7k**). Yield: 46%, oil. ^1^H-NMR (MeOD, 400 MHz) δ: 6.25–6.92 (3H, m, phenyl), 3.72 (3H, s, OCH_3_), 3.70 (3H, s, OCH_3_), 2.13–2.28 (1H, m, N=C-CH_a_), 1.60–1.90 (4H, m, N=C-CH_b_, CH, CH_2_), 1.20–1.55 (2H, m, CH_2_), 1.08 (3H, s, CH_3_), 0.99 (3H, s, CH_3_), 0.90 (3H, s, CH_3_). ^13^C-NMR (MeOD, 100 MHz) δ: 190.60, 155.88, 145.16, 142.30 114.59, 109.98, 108.57, 56.89, 56.18, 55.71, 45.34, 38.31, 33.34, 28.28, 20.08, 19.52, 11.65. IR (KBr) v, cm^−1^: 1682 (C=N). HRMS (ESI, +H): Theoretical mass calculated for [C_15_H_20_N_2_ + H]: 288.1964; Found: 288.1962.

*(E)-N-(1,7,7-Trimethylbicyclo[2.2.1]heptan-2-ylidene)pyridin-2-amine* (**7l**) [33]. Yield: 39%, oil. ^1^H-NMR (MeOD, 400 MHz) δ: 6.75–8.35 (4H, m, phenyl), 2.20–2.55 (1H, m, N=C-CH_a_), 1.70–1.90 (4H, m, N=C-CH_b_, CH, CH_2_), 1.20–1.50 (2H, m, CH_2_), 0.99 (3H, s, CH_3_), 0.94 (3H, s, CH_3_), 0.82 (3H, s, CH_3_). ^13^C-NMR (MeOD, 100 MHz) δ: 185.33, 163.31, 148.45, 137.87 118.99, 115.07, 53.84, 46.83, 43.16, 35.88, 31.49, 26.78, 19.28, 18.78, 11.21. IR (KBr) v, cm^−1^: 1681 (C=N). GC: 100, MS (*m*/*z*, %): 228 (45), 213 (72), 200 (100), 185 (65), 78 (48).

*(E)-N-(1,7,7-Trimethylbicyclo[2.2.1]heptan-2-ylidene)pyridin-4-amine* (**7m**). Yield: 10%, oil. ^1^H-NMR (MeOD, 400 MHz) δ: 6.70–8.45 (4H, m, phenyl), 2.10–2.20 (1H, m, N=C-CH_a_), 1.65–1.92 (4H, m, N=C-CH_b_, CH, CH_2_), 1.20–1.50 (2H, m, CH_2_), 0.98 (3H, s, CH_3_), 0.94 (3H, s, CH_3_), 0.82 (3H, s, CH_3_). ^13^C-NMR (MeOD, 100 MHz) δ: 187.29, 160.06, 149.49, 115.31, 54.22, 43.76, 35.72, 31.64, 26.76, 18.50, 17.89, 15.00. IR (KBr) v, cm^−1^: 1682 (C=N). HRMS (ESI, +H): Theoretical mass calculated for [C_15_H_20_N_2_ + H]: 229.1705; Found: 229.1695.

*(E)-N-(1,7,7-Trimethylbicyclo[2.2.1]heptan-2-ylidene)methyl)thiazole* (**7n**). Yield: 15%, oil. ^1^H-NMR (MeOD, 400 MHz) δ: 7.55 (2H, d, phenyl, *J*=3.6 Hz), 7.35 (2H, d, phenyl, *J*=3.6 Hz), 2.55–2.65 (1H, m, N=C-CH_a_), 2.19 (1H, d, N=C-CH_b_, *J* = 18.4 Hz), 2.06 (1H, t, CH, *J* = 4.4 Hz), 1.85-1.96 (2H, m, CH_2_), 1.30–1.50 (2H, m, CH_2_), 1.08 (3H, s, CH_3_), 1.02 (3H, s, CH_3_), 0.85 (3H, s, CH_3_). ^13^C-NMR (MeOD, 100 MHz) δ: 194.58, 173.30, 140.90, 118.48, 57.07, 45.58, 39.64, 33.11, 28.00, 20.09, 19.45, 11.65. IR (KBr) v, cm^−1^: 1643 (C=N). HRMS (ESI, +H): Theoretical mass calculated for [C_13_H_18_N_2_S + H]: 235.1269; Found: 235.1268.

### 3.6. Synthesis of N-Benzyl-1,7,7-trimethylbicyclo[2.2.1]heptan-2-amine (**8**) [34].

To a solution of the compound **7a** (0.37 g; 1.53 mmol) in methanol (10 mL), NaBH_4_ (0.116 g; 3.06 mmol) was added slowly at room temperature. The reaction was refluxed until TLC analysis indicated total consumption of **7a**. After cooling to room temperature, water (10 mL) was added and the mixture was stirred for 30 min. The solvents were removed by rotary evaporation and the residue was suspended in aqueous K_2_CO_3_ (10 mL, 10% solution) and extracted with ethyl acetate (3 × 10 mL). The combined organic phases were dried over anhydrous Na_2_SO_4_ and evaporated to furnish the crude product which was purified using column chromatography on silica gel (70–230 mesh, AcOEt/n-Hex 10%). Compound **8** was obtained in a 25% yield as an oil. ^1^H-NMR (CDCl_3_, 400 MHz) δ: 7.20–7.35 (5H, m, phenyl), 3.70 (1H, dd, CH_2_Ph, J = 13.0 Hz), 2.59 (1H, dd, CH_2_Ph, J = 13.0Hz), 1.00–1.70 (10H, m, CH-CH_2_, CH, CH_2_-CH_2_, CH_3_), 0.89 (3H, s, CH_3_), 0.81 (3H, s, CH_3_). ^13^C-NMR (CDCl_3_, 100 MHz) δ: 128.45, 128.32, 127.62, 126.88, 66.44, 52.81, 48.67, 46.99, 45.55, 38.98, 37.07, 27.60, 20.83, 20.80, 12.42. IR (KBr) v, cm^−1^: 3067 (NH). GC: 97%, MS (m/z, %): 243 (M^+^, 47), 172 (83), 120 (25), 95 (53), 91 (100).

### 3.7. Synthesis of N′-(1,7,7-trimethylbicyclo[2.2.1]heptan-2-ylidene)isonicotinohydrazide (**9**).

Isonicotinohydrazide (0.28 g; 2.13 mmol) was added to a solution of **1** (0.40 g; 2.13 mmol) in acetonitrile (20 mL). The reaction mixture was refluxed until TLC analysis indicated the consumption of **1**. The precipitate was collected, washed with cold ethanol, and dried under vacuum. The residue was triturated in refluxing ethanol for 2 hours. After cooling at 5 °C, the solid was collected and dried under vacuum to furnish **9**. Yield: 60%, mp: 216–218 °C (lit. [35]: 205–207 °C). ^1^H-NMR (DMSO, 400 MHz) δ: (mixture of isomers *E*/*Z*: 5:1) 10.64 (s, NH, *Z* isomer), 10.48 (1H, s, NH), 8.72 (2H, d, phenyl, *J* = 5.9 Hz), 8.66 (2H, d, phenyl, *Z* isomer), 7.71 (2H, d, phenyl, *J* = 5.9 Hz), 7.59 (2H, m, phenyl, *Z* isomer), 2.55–2.65 (1H, m, N=C-CH_a_), 2.10 (1H, d, N=C-CH_b_, *J* = 17.7 Hz), 1.95 (1H, t, CH, *J* = 4.2 Hz), 1.15–1.90 (4H, m, CH_2_-CH_2_), 0.99 (3H, s, CH_3_), 0.91 (3H, s, CH_3_), 0.87 (s, CH_3_, *Z* isomer), 0.76 (3H, s, CH_3_), 0.70 (s, CH_3_, *Z* isomer). ^13^C-NMR (DMSO, 100 MHz) δ: 178.01, 163.34, 149.48, 148.49 (*Z*), 141.86, 123.78 (*Z*), 121.93, 53.26, 44.08, 43.96, 34.59, 33.66 (*Z*), 32.21, 18.54 (*Z*), 18.43, 17.63, 10.12. IR (KBr) v, cm^−1^: 1638 (C=N). GC: 100%, MS (*m*/*z*, %): 256 (76), 139 (66), 123 (48), 106 (100).

### 3.8. Biological Evaluation against *Mycobacterium tuberculosis*

The antimycobacterial activities of all tested compounds were assessed against *M. tuberculosis* ATCC 27294, using the microplate Alamar Blue assay (MABA, Table 2 and Table 3). Briefly, 200 microliters of sterile deionized water was added to all outer-perimeter wells of sterile 96-well plates (Falcon, 3072: Becton Dickinson, Lincoln Park, NJ, USA) to minimize evaporation of the medium in the test wells during incubation. The 96 plates received 100 μL of the Middlebrook 7H9 broth (Difco laboratories, Detroit, MI, USA) and a serial dilution of the tested compounds was made directly on the plate. The final drug concentrations tested were 3 to 100.0 μg/mL. Plates were covered and sealed with parafilm and incubated at 37 °C for five days. After this time, 25 microliters of a freshly prepared 1:1 mixture of Alamar Blue (Accumed International, Westlake, OH, USA) reagent and 10% Tween 80 were added to the plate and incubated for 24 h. A blue color in the well was interpreted as no bacterial growth, and a pink color was scored as growth. The MIC was defined as the lowest drug concentration which prevented a color change from blue to pink.

### 3.9. Cytotoxicity against Cancer Cell Lines

Compounds (5.0 µg/mL) were tested for their cytotoxic activity against three human cancer cell lines: OVCAR-8 (ovary), SF-295 (glioblastoma), and HCT-116 (colon) (National Cancer Institute, Bethesda, MD, USA). All cell lines were maintained in Roswell Park Memorial Institute (RPMI) 1640 medium supplemented with 10% fetal bovine serum, 2 mM glutamine, 100 U/mL penicillin, and 100 μg/mL streptomycin at 37 °C with 5% CO_2_. Each compound was dissolved with DMSO until reaching the final concentration. The final concentration of DMSO in the culture medium was kept constant, below 0.1% (*v*/*v*). All compounds were incubated with the cells for 72 h. The negative control received the same amount of DMSO (0.001% in the highest concentration). The cell viability was determined by the reduction of the yellow dye 3-(4,5-dimethyl-2-thiazol)-2,5-diphenyl-2H-tetrazolium bromide (MTT) to a blue formazan product as described by Mosmann [36]. Unfortunately, none of the compounds prepared in this study displayed cytotoxicity activities.

## Figures and Tables

**Table 1:** Syntheses and antimycobacterial activity against *Mycobacterium tuberculosis* H37Rv (ATCC 27294) of hydrazones **3**.

Final Compound (3)	R^1^	R^2^	R^3^	R^4^	X	MIC (µg/mL)	Time Classic Method (h)	Yield (%)	Time Ultrasound Method ^a^ (min)
**a**	H	H	H	H	CH	Res	20	64	5
**b**	H	H	F	H	CH	Res	20	80	5
**c**	H	H	Cl	H	CH	Res	20	71	5
**d**	H	H	Br	H	CH	50	20	57	5
**e**	H	H	OH	H	CH	Res	20	70	60
**f**	H	H	OCH_3_	H	CH	100	20	75	5
**g**	H	H	NO_2_	H	CH	ins	20	36	5
**h**	H	Cl	H	H	CH	100	20	70	5
**i**	H	CN	H	H	CH	Res	20	85	5
**j**	H	OCH_3_	H	H	CH	Res	20	61	5
**k**	H	NO_2_	H	H	CH	ins	20	50	5
**l**	F	H	H	H	CH	Res	20	68	5
**m**	Cl	H	H	H	CH	100	20	51	5
**n**	Br	H	H	H	CH	50	20	55	5
**o**	OH	H	H	H	CH	50	20	64	5
**p**	NO_2_	H	H	H	CH	25	20	77	5
**q**	Cl	Cl	H	H	CH	Res	20	78	5
**r**	OH	OH	H	H	CH	100	20	73	7
**s**	OH	H	OH	H	CH	100	20	30	10
**t**	H	OH	OH	H	CH	100	20	38	5
**u**	OH	H	H	OH	CH	Res	20	64	5
**v**	OH	H	OCH_3_	H	CH	50	20	78	18
**w**	OH	H	H	NO_2_	CH	Res	20	54	5
**x**	H	H	H	H	N	100	20	38	5

Res: resistant; ins: insoluble; MIC: minimal inhibitory concentration. ^a^ The yields were very similar for both classical and sonochemistry methodologies.

**Table 2:** Selected bond lengths and angles, Å (°) in **3k**.

C2-N1	1.272(5)	O1-N3	1.218(5)
N1-N2	1.420(4)	O2-N3	1.226(5)
N2-C11	1.268(5)	C11-C12	1.467(5)
N2-N1-C2	112.2(3)	N1-N2-C11	112.0(3)
N2-C11-C12	121.9(4)	O1-N3-O2	123.5(4)
C2-N1-N2-C11	173.4(3)	N1-N2-C11-C12	175.6(3)

**Table 3:** Derivatives camphor **7** from nitroimine **6** and different amines and anilines.

Final Compound (7)	R	MIC (µg/mL)	Condition, Time	Yield (%)
**a**	–CH_2_Ph	Res	CHCl_3_, reflux, 4 h	55
**b**	–CH_2_(CH_2_)_2_CH_3_	Res	CHCl_3_, reflux, 4 h	70
**c**	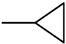	Res	CHCl_3_, reflux, 3 h	65
**d**	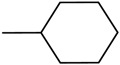	Res	CHCl_3_, reflux, 3 h	73
**e**	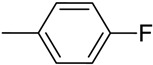	Res	CH_3_CN, reflux, 48 h	79
**f**	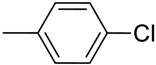	100	CH_3_CN, reflux, 30 h	52
**g**	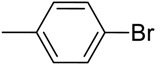	100	CH_3_CN, reflux, 43 h	73
**h**	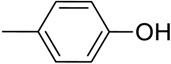	Res	CH_3_CN, reflux, 17 h	81
**i**	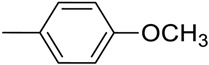	Res	CH_3_CN, reflux, 48 h	90
**j**	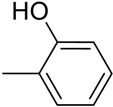	3.12	CH_3_CN, reflux, 20 h	53
**k**	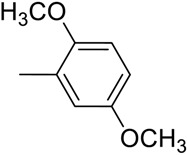	Res	CH_3_CN, reflux, 48 h	46
**l**	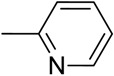	Res	CH_3_CN, reflux, 64 h	39
**m**	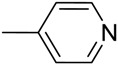	Res	CH_3_CN, reflux, 24 h	10
**n**	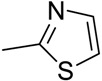	Res	CH_3_CN, reflux, 72 h	15
